# Exploring the contribution of social capital in building resilience for climate change effects in peri-urban areas, Dar es Salaam, Tanzania

**DOI:** 10.1007/s10708-020-10214-3

**Published:** 2020-05-20

**Authors:** Lazaro Eliyah Mngumi

**Affiliations:** 1 Institute of Human Settlements Studies (IHSS), Ardhi University, P.O. Box 35176, Dar es Salaam, Tanzania; 2 Faculty of Natural Resources and Agricultural Sciences, Swedish University of Agricultural Sciences (SLU), P.O. Box 7012, 750 07 Uppsala, Sweden

**Keywords:** Bonding social capital, Bridging social capital, Peri-urban areas, Climate change resilience building, SSA

## Abstract

This article aims to contribute to the literature on the quest for resilient cities by focusing on the climate change resilience building discourse in peri-urban areas, and specifically by exploring the role of social capital-an under-researched topic. The article examines bonding social capital and bridging social capital, with a focus on how they can potentially contribute to, or inhibit, the socio-ecological system resilience building processes in the context of climate change reality in peri-urban areas. Theoretically, the author draws on the existing social capital and resilience related literatures; empirically, the article presents findings from a study conducted in the peri-urban areas of Pugu and Kazimzumbwi forest reserves on the outskirts of Dar es Salaam city in Tanzania. The study deployed a household survey and key informant interviews. It found that both bonding and bridging social capital were strong in the research area, suggesting the feasibility of building resilience to climate change effects. Examples are given of a number of resilience building interventions that were established through synergies between social capital actors and local communities, although some doubt is cast over the sustainability of these initiatives. Overall, both theoretical and empirical evidence suggests the importance of including a focus on social capital in exploring the building of climate change resilience pathways in peri-urban areas, and especially in the context of the global south.

## Introduction

The quest for resilient cities is becoming increasingly urgent amongst policy makers and the academia across the world. As complex entities, cities provide multiple entry points through which resilience can be explored and negotiated. These already complex pathways to resilience are further complicated by the issue of climate change in the developing world, particularly in Sub-Saharan Africa (SSA). The reality is that, while cities in the developing world are experiencing some of the highest risks of global climate change, they are largely ill-equipped to adapt to the changing climatic conditions. This article aims to contribute to the subject of the quest for resilient cities by exploring the climate change resilience building discourse, and more specifically the social capital aspects pertinent to that discourse, in the peri-urban context of SSA. Explicitly, the article explores social capital with a focus on how it can potentially contribute to or inhibit socio-ecological system resilience (SESR) building processes in the context of climate change. It examines two major forms of social capital, i.e. bonding and bridging, and discusses their potential impact on climate change resilience discourse in the peri-urban developing world context. To realize this aim, the article engages in both conceptual and empirical exploration. While the theoretical exploration is drawn from social capital and resilience related literature, the empirical information comes from research in the peri-urban areas of Pugu and Kazimzumbwi forest reserves (PKFRs), on the outskirts of Dar es Salaam city in Tanzania. Moreover, the article builds upon and complements the growing number of studies on these peri-urban forest reserves which have established the continued decline of ecosystem services (Lupala et al. 2014; Lupala and Maglan 2015; Kashaigili et al. 2013, 2014). These studies have linked the reduction of ecosystem services to climate change effects as peri-urban dwellers resort to encroaching on the reserves as part of their autonomous adaptation strategy. This has led to a call to explore the potential for building sustainable SESR. Recent research work by Mngumi (2019) in ‘these’ peri-urban forest reserves has in part answered this call, suggesting that ecosystem services-based adaptive capacities offer enormous potential for building resilience against climate change effects. The work by Mngumi contributes to filling the knowledge void on the ecological dimension identified in earlier studies; it also highlights the need to explore the social aspects necessary for building resilience to climate change effects in these peri-urban areas. It is against this background that the present article contributes to furthering the scholarship in this topic, which is under-researched in general and especially in the context of peri-urban areas in SSA. Following this brief introduction, the article comprises four sections. The next section offers a theoretical review, while “Materials and methods” section describes the methodology of the study. The major empirical findings are presented and discussed in “Results and discussion” section, while “Conclusions” section highlights the main conclusions of the study and makes some suggestions for further scholarship.

## Theoretical review

This section provides the conceptual framework for the topic of the article, i.e. the potential contribution of social capital to SESR building in the context of climate change effects in peri-urban areas. Whilst the scholarship on climate change resilience has been growing broadly, it can be argued that the focus has been skewed, particularly in the global south. First, there has been a focus on an ‘engineering resilience’ perspective, at the expense of the SESR perspective. Second, there has been relatively little attention in climate change resilience research to peri-urban spaces. This section will examine these two apparent biases in climate resilience research, thereby highlighting the conceptual topicality of this article. It will also provide an analysis of the two categories of social capital which form an integral part of the article—bonding and bridging social capital—and elucidate how they might potentially contribute to or inhibit SESR building for climate change effects in peri-urban areas.

The concept of resilience has been variously defined across disciplinary and geographical contexts. Originating from physical sciences, resilience is used to describe the capacity of material or systems to return to their normal state, or equilibrium, after a displacement (Norris et al. 2008; Bodin and Wiman 2004). In the original usage of the term, a material is said to be resilient or to have resilience when it bends and bounces back rather than breaking when subjected to stress (Bodin and Wiman 2004). System resilience, meanwhile, depends upon one constituent of a system being able to change or adapt in response to changes in other constituents; without this adaptation, the system would fail to function (Adger 2000). This type of resilience is known as engineering resilience (Norris et al. 2008; Gunderson 2000). This approach to resilience has been dominant in the context of climate change resilience in SSA cities including Dar es Salaam—a dominance which is rationalized in part by what is known as the infrastructural deficit challenge (IDC). In much of the developing world, the IDC is a lived reality which dictates to a considerable extent the urban planning and development processes. This focus on IDC and engineering resilience has largely jeopardized the deployment of a social lens in exploring and understanding social issues pertinent to resilience building discourses in urban and, especially, in peri-urban contexts in SSA. Yet, resilience is increasingly understood to be largely a social phenomenon. The social nature of resilience is premised upon the idea that resilience is a process of navigating plausible potential options towards envisaged positive futures and is not an end in itself. At the core of this ‘notion’ lies the imperative of contextualizing resilience, i.e. setting resilience within its social milieu. While this article focuses specifically on the role of social capital in building SESR in relation to climate change effects in peri-urban areas in the global south, it is worth noting that the social milieu which encompass resilience-building discourses are much broader than social capital. Other intriguing social discourses pertinent to SESR-building processes include those around agency, sustainability, power and power relations.

However, as hinted beforehand, climate change resilience scholarship in peri-urban contexts has not received a lot of attention. Indeed, there are challenges involved, especially in the context of the developing world, owing to lack of consensus around the concept of the peri-urban in academia and among development practitioners (Thuo 2013; Forsyth 2012). The concept is deployed in this article not primarily with the aim of engaging in that debate; rather, this conceptual contestation is reviewed so as to facilitate a deeper understanding of the social attributes of the peri-urban context, as part of the exploration of SESR in response to climate change effects. In brief, there is increasing agreement within academia on the lived realities of the diverse, context-laden definitions of the peri-urban concept (Mbiba and Huchzermeyer 2002; Salem 2015) as well as the co-existence of urban and rural features within cities and beyond their limits (Salem 2015; Allen et al. 2006). Inspired by this convergence of understandings, and taking account of the physical context of this research, this article conceives the ‘peri-urban’ as a city’s transitional zone, amalgamating the functions and features of both urban and rural landscapes. More explicitly, in the presentation of empirical information in this article, ‘peri-urban’ is used to connote the ‘Pugu and Kazimzumbwi socio-ecological system’ (PK-SES), located on the outskirts of Dar es Salaam, which is one of the most rapidly growing cities in Africa and the current business capital of Tanzania. Similar to other peri-urban areas in SSA (Roy et al. 2017; Mngumi 2020), ecosystem services at PK-SES are on the decline. At the same time, climate change resilience efforts in the SSA region are increasingly skewed towards urban areas, leaving peri-urban areas largely overlooked. One of the factors behind this neglect of peri-urban areas is the issue of inadequate infrastructure. This IDC can be considered the result of a systemic failure, exacerbated by the unprecedented population growth linked to rapid urbanization under poverty. In other words, the limited theoretical attention and practical intervention focused on peri-urban areas can be explained in part by the general planning quagmire which affects such areas. Moreover, while peri-urban spaces are largely neglected, there is a growing recognition amongst scholars (Jones et al. 2012; Rosenzweig et al. 2018; Jones et al. 2012) and policy makers (UNHABITAT 2015; UNFCCC 2017) that they can potentially offer low-cost options for building climate change resilience for cities and their wider regions. This growing understanding presents an opportunity that cities of the developing world can capitalize upon in their quest for climate resilient socio-ecological systems, which would by default contribute towards realizing resilient cities. Ecosystem services are a key aspect of the notion that peri-urban spaces are imbued with low-cost potential for building SESR for climate change effects in cities. It is beyond the scope of this article to discuss all categories of ecosystem services: rather, it focuses on two—the cultural and the provisioning ecosystem services. This article is a follow-up of the work already published (Mngumi 2019), and aims to advance scholarship in this relatively new area of study, looking at the role of social capital in the context of climate change resilience in SSA, and specifically in the peri-urban areas of Dar es Salaam.

### Social capital perspectives in climate change resilience-building discourse

The understanding and application of the concept of social capital is still evolving. Bourdieu (1986, p. 248) offered the first definition of social capital, as ‘the aggregate of the actual or potential resources linked to possession of a durable network of more or less institutionalized relationships of mutual acquaintance and recognition’. He argued that social networks are not given but rather constructed through investment strategies oriented to the institutionalization of group relations, usable as a reliable source of benefits. The concept of social capital revolves around the idea that well-connected community members are better positioned to mobilize resources in pursuing their desired outcomes (Agnitsch et al. 2006). Social capital is thus built up by norms and networks that enable people to act collectively (Woolcock and Narayan 2006). Woolcock and Narayan argue further (idem.) that despite being articulated differently across disciplines, the idea that social networks oil the wheels of collective action is intuitively appealing in resilience-building discourse. Adger (2010) similarly argues that social capital has the potential to serve as an essential ‘catalyst’ for fueling economic development. In other words, the presence of social capital increases the capacity for action and the realization of wider community goals. This suggests that social capital has the potential to contribute towards achieving a myriad of development outcomes, especially at the community scale.

Although widely used in other development contexts, social capital has not been very prominent in climate change studies. However, seeing the impact that social capital has had on economic development and well-being (Ostrom 2000), there is a growing acknowledgement of its inherent potential in building SESR against the negative effects of climate change. The role of social capital is likely to be even greater when SESR building is explored in the developing world, given that the imperatives of social capital are even stronger in contexts of poverty—for instance, social connectedness, help in times of need, and social safety nets—especially when poverty is exacerbated by additional shocks and stresses such as climate change. Moreover, social capital is increasingly regarded as a platform upon which other forms of capital—such as agency, innovative economy—can be effectively developed in building resilience to climate change (Agnitsch et al. 2006). Yet, despite the increased understanding of the potential role of social capital on resilience building, its application in the urban climate change context is negligible, as most interventions still invest heavily in physical infrastructure (Adger 2010; Aldrich and Meyer 2015).

Two categories of social capital are particularly relevant for this article: bonding social capital and bridging social capital (Aldrich and Meyer 2015; Agnitsch et al. 2006; Putnam 2000). Putman provides an elaborate description of these categories of social capital. Bonding social capital looks more inwards and emphasizes identities and groups of a homogeneous nature (Putnam 2000). Putman explains that bonding social capital thrives in well-embedded groups with strong affective ties linking group members to one another, and is crucial in providing social support and in cementing in-group solidarity. This type of social capital is important in building socio-ecological system resilience in climate change affected communities in peri-urban areas of the developing world: this is because social support and in-group solidarity are crucial in preparing the ground for other adaptive capacity attributes necessary to build socio-ecological resilience to climate change effects.

However, the co-existence of urban and rural features in peri-urban areas of the developing world makes deployment of bridging social capital even more imperative in building SESR. Bridging social capital links people or groups of different orientations; in addition, it addresses how social capital facilitates resource acquisition (Putnam 2000), which is central to resilience pathways. Unlike bonding social capital, whose networks are comprised of similar individuals with supposedly equivalent resources, bridging social capital can address the array of differences typical of peri-urban areas. It also plays a crucial role in facilitating information flow within and between groups and improving access to a wide range of resources (Ibid.). Bridging social capital enables the best use of relevant knowledge and technical know-how in terms of what is feasible and what options have most potential in a given context. The role of this category of social capital in building SESR against climate change effects in peri-urban areas of the global south is related to the realities of poverty linked to resource scarcity. This reality renders sections of the population at odds with each other when subjected to shocks and stresses such as climate change. If left on their own without the external synergies made possible by bridging social capital, communities would devise their own autonomous adaptation modalities which would most likely undermine long-standing efforts geared at addressing climate change effects. This scenario has indeed manifested itself in the peri-urban areas of PK-SES. After being subjected to climate change which reduced their livelihood options, a substantial proportion of community members resorted to encroaching on adjacent ecosystem services to earn their living, with no regard for the impact of their actions on those ecosystem services and on the climate. This undermined earlier initiative towards addressing climate change by, for example, degrading forest ecosystems, thereby reducing the carbon sequestration function of those forests. The ‘resource gap’ that is thus created can be at least partly addressed by bridging social capital. How the synergies of bridging social capital were manifested in the peri-urban areas of PK-SES will be articulated in the results and discussion section below.

However, like all theories, social capital has its critics. Most criticisms are leveled against the overly positive effects attributed to bonding social capital. Scholars have pointed out some of the negative effects of close-knit, trusting groups, which include the exclusion of outsiders, excess claims on group members, restrictions on individual freedoms, and downward leveling norms. At the same time it is increasingly agreed that street gangs, mafia groups, drug rings, and racial supremacy groups are all likely to feature high levels of bonding social capital, yet their actions often lead to undesirable and harmful outcomes (Agnitsch et al. 2006). If this unfolds, it would likely jeorpadise the efforts towards building SESR in peri-urban areas.

## Materials and methods

As mentioned beforehand, this paper builds upon previous research exploring SESR for climate change effects in peri-urban areas of Dar es Salaam. The initial study explored the SES adaptive capacities imperative in building SESR for climate change effects in the peri-urban areas of the PKFRs. This initial research revealed important cultural and provisioning ecosystem services with potential for building resilience to climate change effects in these peri-urban areas. These included both cultural adaptive capacities (socio-ecological memory through diverse age cohorts, promising literacy rate, and diverse ethnic groups), and innovative economic capacities (such as bee keeping, as well as various forms of tourism related to arts and crafts, local food, wildlife, eco/green tourism, and antiquities tourism). This paper extends that research in the same geographical setting, by focusing on social capital—another crucial element in building SESR for climate change effects in peri-urban areas and particularly in the SSA context. Given the limited research on this topic to date, this article contributes both conceptually and empirically to the current literature. This section describes the study area and the methods employed.

### Study area description

**Fig. 1:**
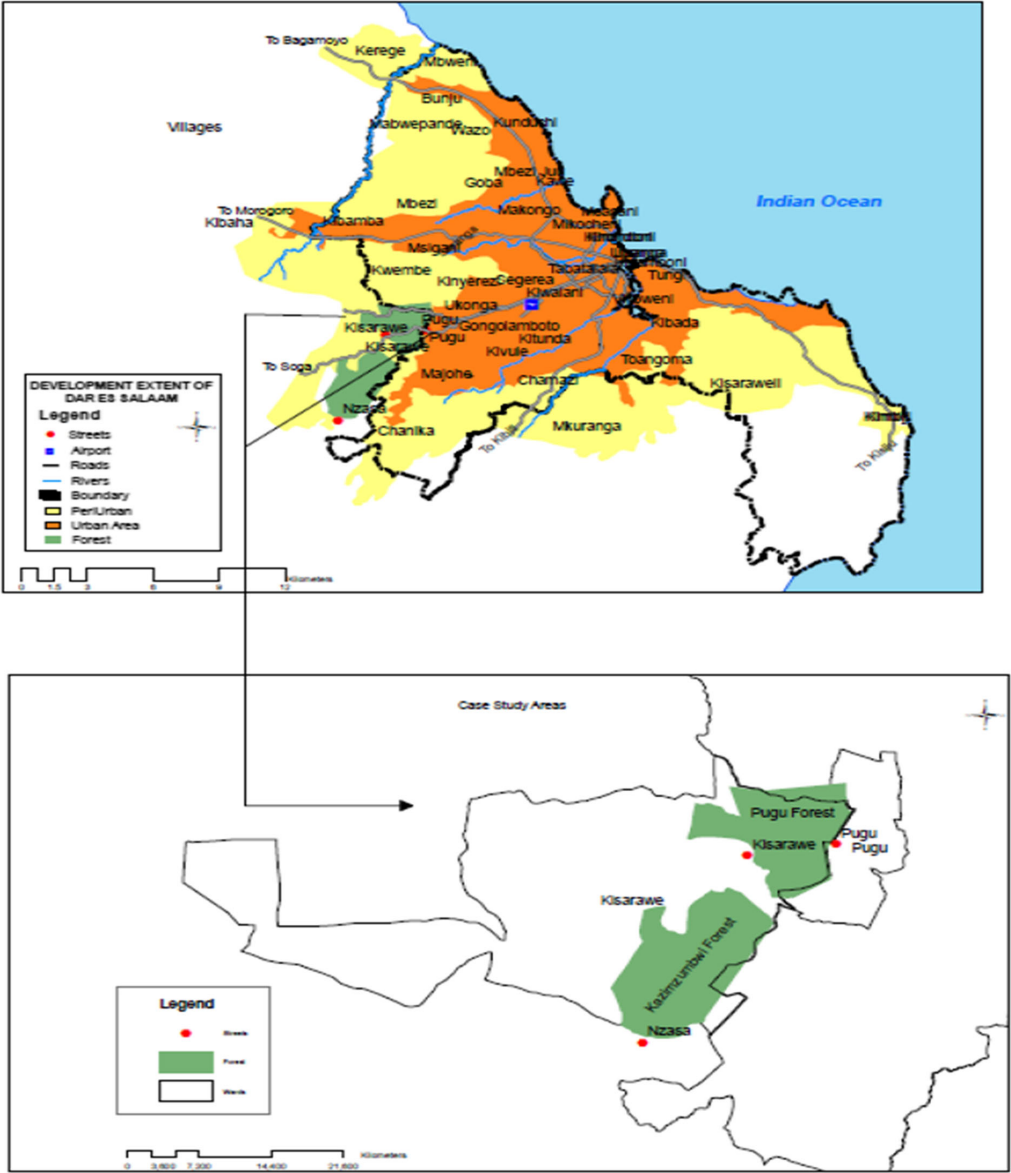
The study area (PKFRs) at the peri-urban belt of Dar es Salaam. *Source*: Erimina Massawe; Ardhi University (2019)

The Pugu and Kazimzumbwi forest reserves (PKFRs), which lie within the peri-urban belt to the southwest of Dar es Salaam city in Tanzania (Fig. 1), form the setting for the case study. Administratively, the PKFRs are largely under the jurisdiction of the Kisarawe District Council (KDC) in the Coast Region of Tanzania, although a limited portion of Kazimzumbwi forest reserve falls within the administrative boundaries of the Ilala District in Dar es Salaam Region. The PKFRs are surviving remnants of some of the world’s ancient forests; they are of global importance as they support 37 endemic vertebrate species and about 554 endemic plant species (Burgess 2000). Accordingly, they offer important insights for this study as the ecosystem services they provide contribute to our understanding of SESR building for climate change effects in peri-urban areas. The PKFRs also form the catchment area for a number of rivers: Msimbazi, Mambizi, Mzumbwi, Vikongoro, Kimani, Nzasa and Nyeburu (Lupala et al. 2014; TFCG 2013). The Pugu forest, which was gazetted as a reserve in 1954, lies in the northeastern part of the Pugu Hills, about 25 km from the Indian Ocean. The Kazimzumbwi forest reserve, which was gazetted in 1936 (Clarke and Dickinson 1995), lies to the southwest of Pugu. Both forests are influenced by the Indian Ocean tropical monsoon climate: they are characterized by a bimodal rainfall pattern which brings long rains between late March and early June, popularly known as Masika, and short rains between October and December, known as Vuli. The average annual rainfall is approximately 1,100 mm, and the temperature ranges from 24° to 31° depending on elevation (Clarke and Dickinson 1995; Lupala and Maglan 2015). Topographically, Pugu forest is positioned between 100 and 305 m above sea level, while Kazimzumbwi forest lies between 120 and 280 m above sea level (TFCG 2013).

The study was conducted in three hamlets, two—Pugu-Kibaoni and Kisarawe—located adjacent to the Pugu forest reserve and the third—Nzasa—located adjacent to the Kazimzumbwi forest reserve (Fig. 1). The three study hamlets were purposively selected in order to include a hybrid mix of social capital in the PKFRs, reflecting the typical representation of peri-urban characteristics in SSA i.e. the co-existence of urban and rural features. Although all three hamlets can be classified as peri-urban, Pugu-Kibaoni lies close to the urban part of the city, while Kisarawe and Nzasa hamlets are located in more rural areas and tend to display more rural than urban characteristics.

### Methods

Data for this study were collected through key informant interviews and a household survey. The two methods were deployed sequentially; key informant interviews were undertaken first, followed by household surveys. This sequence was important as key informant interviews laid the ground for the deeper understanding of the broader social capital milieu, with the specific focus on SESR for climate change effects. Moreover, the key informant interviews yielded information that enabled fine tuning of the household questionnaire which was later deployed for the purpose of mapping social capital in the PK-SES. Whilst I was personally involved in carrying out interviews with key informants, the household survey was executed by three research assistants. These three enumerators were university graduates, which was necessary to ensure that they could easily understand and follow the data collection protocol and adhere to the prescribed ethical standards. Another driver that prompted the use of graduates in collecting data was the fact that the household questionnaire was framed in English (see “Appendix”), so it was essential to use enumerators who were fluent in English and Swahili. Prior the execution of the survey, I convened a two-day training session to familiarize the enumerators with the survey questions, and the entire survey protocol. Since the educational level of the majority of respondents was primary or lower (see Table 1), and some could not read or write well, the enumerators were responsible not only for asking the questions but also for filling in the responses on the questionnaires. The household survey took place over two consecutive weeks, with one enumerator responsible for each of the three hamlets. Every evening, I convened a brief session with all the enumerators to cross check the filling of the questionnaires and to receive feedback on the progress of the survey. This forum was used for discussing and deliberating upon any emerging anomalies from the survey work. This was crucial for ensuring data quality and consistency of the collection process, which is an integral part of the internal validity check for data quality in a case study inquiry. Once submitted, the completed questionnaires were kept in the case study database pending further scrutiny and analysis.

**Table 1:** Selected characteristics of the study participants. *Source*: Field work analysis 2019

Respondent’s characteristics	Male (N = 91)		Female (N = 57)		Total (N = 148)	
	Mean	SD	Mean	SD	Mean	SD
Age (years)						
< 30	25	3	24	4	24	4
30+	45	14	51	14	48	14
All	35	15	40	17	37	16

	Male	%	Female	%	Total	%
Education status						
Primary or less	60	41	44	30	104	70
Secondary	21	14	9	6	30	20
High school	2	1	2	1	4	3
Certificate and above	8	5	2	1	10	7
Residence status						
Native	26	18	22	15	48	32
Migrant from DSM	25	17	25	17	50	34
Migrant from upcountry	40	27	10	7	50	33.8
Geographic location						
Nzasa	23	16	27	18	50	33.8
Pugu-Kibaoni	37	25	12	8	49	33.1
Kisarawe	31	21	18	12	49	33.1

A total of 50 questionnaires were randomly administered in each of the study hamlets, amounting to 150 in total for the study area (PK-SES). The sample size representation is approximately equivalent to 10% of households per hamlet, which is recommended for statistical analysis. Of those approached, just over 98% agreed to participate in the survey, resulting in 148 completed surveys. Of these 148 respondents, 57 were females and 91 were males; 48 were natives of the peri-urban area, 50 were migrants from Dar es Salaam and 50 were migrants from upcountry. As indicated above, the three hamlets were chosen for their slightly different socio-economic characteristics within the peri-urban setting of the PK-SES, in order to assess social capital of populations of diverse backgrounds. Study households were randomly selected from the residential areas and only one participant was involved per household. The minimum age for participation was 17 years of age, but priority was given to household heads. Participants ranged from 17 up to 73 years of age; the average age was 37 with a standard deviation of 16. The participants’ main characteristics are summarized in Table 1.

The household survey was used to assess social capital attributes i.e. bonding and bridging forms of capital, following the standard example questionnaire of (Chen et al. 2008). The questionnaire consisted of ten scales/Cap^1^ with 42 sub-items. Survey analysis was carried out using principal component analysis (PCA), through the statistical package for social sciences (SPSS), version 24. Explicitly, the 42 sub-items were assessed in a five-point Likert scale with *One *= ‘none’ or a few and *five *= ‘all’ or a lot. Scores for the individual 10 items were obtained by calculating the summary of constituent sub-item scores and then dividing by the number of sub-items in that particular scale. For instance, the first scale was composed of six sub-item scores for assessing the size of social network connections. The score of this scale was obtained by first taking the six sub-item scores and then dividing the subtotal by six *(the number of sub*-*items*). After the item scores were derived, bonding social capital was obtained by adding together the first five scales (*one* to *five*) and dividing by *five* (the *number of items*). Bridging social capital was calculated by adding together the last five scales (*six* to *ten*) and dividing by five (*the number of items*). The total social capital score was then obtained by adding together the bonding social capital and bridging social capital scores.

The key informant interviews were deployed to acquire a deeper understanding of the social capital milieu at the PK-SES, and were explicitly tailored to capture bridging social capital (which is increasingly known as linking social capital). The key informant interviews were geared towards exploring the links and networks involved in building SESR for climate change effects within the peri-urban context of the PK-SES, especially those related to provisioning and cultural ecosystem services. In other words the interviews were focused specifically on social capital links and networks related to the potential innovative economic options mentioned above, i.e. the bee keeping industry and multiple forms of tourism (Mngumi 2019). A total of 27 interviews were undertaken amongst 20 social capital networks (or social capital nodes); in some social capital networks, more than one representative was interviewed (see Table 2). The peri-urban bridging social capital networks involved in SESR for climate change effects were identified through a snowball sampling technique i.e. bridging social capital players were earmarked via previous interviews (see Mngumi 2019). The social capital networks involved in the interviews can be categorized into three broad groupings: government-based networks; non-government based i.e. non-governmental organizations (NGOs) and companies; and community-based agents (CBAs). Table 2 provides the mapping of the social capital network players interviewed.

**Table 2:** Linking social capital players. *Source*: Field work 2019

S/n	Player category	Player name	Number of interviews
1	Government	Tanzania Forest Services (TFS)	5
2		KDC	3
3	NGOs/Companies	Afri Roots	1
4		Green voices	1
5		Wildlife Conservation Society of Tanzania (WCST)	1
6		Tanzania Forest Conservation Group (TFCG)	1
7		Jane Goodall	1
8		Fly Company	1
9		Pugu Hills Cultural Tourism Enterprises (PHCTE)	1
10		Kisarawe Cultural Tourism Enterprises (KCTE)	1
11		Minaki Hills Cultural Tourism Enterprises (MCTE)	1
12		Safari 56	1
13	Community-based	Popote Pamoja Sikuzote	2
14		MJUMITA	1
15		Uhifandi wa Misitu Tarafa ya Sungwi	1
16		Umoja wa vijana	1
17		Mwangaza	1
18		Nchage	1
19		Vigama	1
20		Maguruwe	1

## Results and discussion

This article aims to provide a discussion amalgamating both empirically grounded information from the PK-SES and theoretical insights emerging from the research, in order to contribute at both the empirical and conceptual levels to an understanding of the role of social capital in SESR in SSA, peri-urban context. To allow for a deeper analysis, this section offers separate but complementary discussions of the two forms of social capital—bonding and bridging.

### Bonding social capital and its potential contribution towards building SESR in peri-urban areas in SSA

As hinted beforehand, bonding social capital was largely explored in this research through a household survey. The findings on bonding social capital in the peri-urban areas of PK-SES are presented in Table 3, and show the Cronbach’s alpha score^2^ for bonding social capital to be 0.70. Given that the Cronbach’s alpha scale ranges from 0 to 1 (Gliem and Gliem 2003), a score between 0 and 0.5 connotes a low degree of embeddedness of the community under investigation. Conversely, a score between 0.5 and 1 indicates strong social bonds or close social ties in the community in question. Thus, the score of 0.70 for the research site suggests that the peri-urban communities in the study have relatively strong social ties.

**Table 3:** Bonding and bridging social capital. *Source*: Field data analysis 2019

S/n	Cap 1-10	Scores	Social capital
1	Cap 1	0.72	(0.70) bonding capital
2	Cap 2	0.60	
3	Cap 3	0.64	
4	Cap 4	0.67	
5	Cap 5	0.88	
6	Cap 6	0.94	(0.83) bridging capital
7	Cap 7	0.63	
8	Cap 8	0.87	
9	Cap 9	0.94	
10	Cap 10	0.77	

The level of bonding social capital demonstrates the substantial degree of connectivity among the members of the peri-urban community in the PK-SES. This indicates the existence of a high level of internal cohesion that can be used in the course of building SESR for climate change effects. The role of bonding social capital in building SESR lies in its potential influence on economic development which is a vital component in the resilience building discourse. In particular, bonding social capital is regarded as an essential element for collective action which is a key ingredient for SESR building processes. The importance of social capital in supporting economic development which can promote SESR building processes in the face of climate change effects in peri-urban areas in SSA is twofold. The first aspect relates to the widespread income poverty experienced in SSA, which highlights the crucial role played by bonding social capital: close social ties and help in times of need are of paramount importance in navigating pathways for building SESR in general, but also with specific reference to peri-urban areas which are subject to stress, including climate change stress. Building SESR is a key way of addressing and alleviating this stress, and social capital lies at the core. As previously noted, this holds true for SSA and for other countries with similar social and economic structures in the global south which face widespread poverty linked to community dependence on (unsustainable) extraction from provisioning ecosystem services (Mngumi 2019). This extractive dependency—or socio-ecological interdependence—highlights the reality of poverty in these communities. It also demonstrates the importance of deploying bonding social capital as an important tool in building SESR against climate change effects. This is very clear in the study area which is characterized by poverty and experiencing the negative impact of climate change on ecosystem services. Bonding social capital is thus increasingly regarded as an essential stimulus for fueling economic development, thereby forming a key attribute in building SESR against climate change effects in peri-urban areas.

The second aspect is the role of bonding social capital in paving the way for other adaptive capacities for building peri-urban SESR against climate change effects. The following sub-section will detail the attributes of bridging social capital in building SESR at the PK-SES, but at this juncture it is important to note the role of bonding social capital as an anchor for other adaptive capacities, since all activities have to be channeled through the existing social capital structures. In this case, existing social ties and networks serve as primary nodes for accessing and disseminating the newer resources such as technology or information essential for building SESR against climate change effects. The effectiveness of the supportive role of bonding social capital in creating a conducive environment for peri-urban SESR building is dependent on the degree of bonding social capital itself. In general, the higher the level of bonding social capital, the greater the potential for other factors to contribute effectively towards building SESR. However, whilst strong social ties are considered to be of considerable importance in building SESR against climate change effects, there are limits: when bonding social capital becomes extremely strong (a Cronbach’s alpha score of close to 1), it is likely to result in groups that are so powerful as to be damaging (Agnitsch et al. 2006). When this happens, the strength of social ties and networks might actually jeopardize SESR building pathways. This would necessitate exploring modalities for offsetting the bonding effect towards building SESR. As Table 3 shows, the bonding social capital score for the peri-urban areas of PK-SES is strong but not extreme; there is therefore no likelihood of jeopardizing the process of building SESR. Moreover, the damaging effect of strong bonding capital is context and case specific. In some scenario it has actually turned out to be of an anticipated help in crisis. This has been recently evidenced in South Africa where the cronic harmful gangs have been reported to support distribution of food and other humanitarian services to the poor households following the recent Covid-19 pandemic lockdown strategy.

### Bridging social capital and its bearing on peri-urban SESR building in SSA

As noted, the empirical data on bonding social capital were largely gathered through a household survey; whereas information concerning bridging social capital was gathered through the household survey but complemented by the key informant interviews.

The household survey results presented in Table 3 depict a bridging social capital score of 0.83 on the Cronbach’s alpha scale. As explained above, this is a strong score, indicating the existence of substantial bridging social capital in the peri-urban areas of PK-SES. Given the potential of bridging social capital in building SESR against climate change affected peri-urban SES in SSA, the Cronbach’s alpha scale score in the study area is promising. Like many other peri-urban areas in SSA, the PK-SES sites are experiencing declining ecosystem services, with negative implications for livelihood options and for climate change adaptation and mitigation potential. Indeed, ecosystem services decline in the PK-SES is likely to have been partly triggered by climate change effects, forcing peri-urban dwellers to increasingly exploit local ecosystem services as a way of adapting to the growing effects of climate change. Although such autonomous adaptation may not be bad in itself, when viewed through the lens of climate change, it can become critical. This raises the question of soliciting and engaging input which is external to the SES in question in order to support local potential in building SESR against climate change effects to arrive at a ‘win–win’ outcome. The sum of resources external to the SES which synergistically engage with existing potential within the SES towards building SESR, is expressed as bridging (or linking) social capital. As already noted, analysis of the household survey suggests that the PK-SES has considerable bridging social capital resources, or resilience-building factors (adaptive capacities). As Table 3 shows, bridging social capital is particularly strong, relative to bonding social capital. While, as afore-hinted, very high levels of bonding social capital can be undesirable, this is not the case for bridging social capital; here, the higher the score the better in terms of building synergies, integrating resources from inside and from outside the SES.

In this study, key informant interviews complemented the information collected via the household survey on bridging social capital in the peri-urban areas of PK-SES. The key informant interviews broadened the space for deeper exploration and understanding of the potential within bridging social capital for building peri-urban SESR against climate change effects in SSA context. It was through key informant interviews that distinctive interventions integrating resources towards building SESR were examined. For the purposes of this research, the bridging social capital being studied was limited to that pertaining to cultural and provisioning ecosystem services.

Bridging social capital networks were established at the PK-SES by a range of players, as portrayed in Table 2 (above), each playing a substantial role towards building SESR against climate change effects. These integrative networks embodied ‘alien’ thinking (from outside the PK-SES) which was, however, crafted within local structures and which deployed local resources towards the common goal of building SESR. The formation of such networks was repeatedly noted during key informant interviews. One such network comprises the village environmental committees (VECs). In an interview with one TFS official, the formation of VECs was described as follows:

Through the influence of WCST, VECs were formed in villages adjacent to the PK-SES. These committees have been instrumental in establishing tree nurseries which have increased income to community members and thereby reduced the dependence on forest reserve for survival. The committees have also provided valuable contribution in reducing the level of encroachment to these peri-urban forest reserves.

From key informant interviews it could be deduced that the formation of VECs in hamlets surrounding the PK-SES was having a positive effect. Thanks to WCST, a number of such committees were formed in hamlets including Kisarawe, Maguruwe, Vibula, Chanika, Nzasa, Nyeburu and Vigama. VECs were partly responsible for raising community awareness regarding the importance of conserving PKFRs, highlighting the benefits accruing to the community as well as those accruing to the environment, specifically in the context of climate change mitigation and adaptation. Through VECs, tree nurseries were established which played two important roles: firstly, they served as a source of income to the members of the VECs; secondly, they provided a continuous source of tree seedlings. This in turn gave an impetus to tree planting campaigns undertaken by WCST and KDC which aimed to promote tree planting within the boundaries of the PKFRs and beyond. Tree planting in areas adjacent to the PKFRs promoted village forest reserves which in turn serve as a buffer zone for the main forest reserves. This has not only reduced dependency on provisioning services in the PKRFs but has also broadened community livelihood options. Thus, the formation of the VECs had a tangible impact on the peri-urban dwellers and also contributed towards building SESR against climate change effects.

Another key role for bridging social capital in the context of the PK-SES was the promotion of climate friendly income-earning activities—i.e. livelihoods which do not exacerbate climate change but rather help to mitigate against it. This provides community members with survival strategies which reduce the level of forest encroachment and its negative consequences vis-à-vis climate change (Scott and Becken 2010). This was partly achieved through the concerted efforts between bridging social capital ‘players’ and resources inherent within the PK-SES. Activities geared towards promoting climate friendly income generation included bee keeping, introduced and championed by a number of the governmental and NGO groups (TFS, KDC, WCST, Green Voices) and community-based groups (PPS, Mwangaza, Vigama, Maguruwe, Nchage and Chanzige). Bee keeping was thus introduced through bridging social capital external to the PK-SES (WCST and Green Voices); newly established bee keeping groups were supplied with the initial capital necessary for start-up, which was particularly vital as most members of the groups could not raise enough funds for intalling the project due to income poverty. This initial financial help was followed by capacity building, assisting the groups with establishing bee hives, hive management, bee products and their respective potential markets. This example supports the theoretical preposition elucidated in “Social capital perspectives in climate change resilience-building discourse” section, that social capital is an important factor in building SESR in peri-urban areas of the developing world and SSA in particular. While the argument in “Social capital perspectives in climate change resilience-building discourse” section relates to bonding social capital, via its collective action potential, the fieldwork shows that bridging social capital was also vital in the PK-SES, suggesting that this type of social capital can play a synergistic role in building SESR in peri-urban in SSA. In this context, bee keeping, as a climate friendly economic activity (provisioning ecosystem service), enabled peri-urban dwellers to increase their income, which in turn reduced the potentially damaging community dependence on other, at-risk ecosystem services. Given the potential benefits of bee keeping, the responsible authorities felt justified in allowing such activities to take place within the forest reserves.

Another important climate friendly income earning activity derived from bridging social capital is the planting of early maturing hybrid mango tree species (EMHMTS). This initiative was also driven by WCST, which distributed EMHMTS to peri-urban dwellers at the PK-SES for commercial purposes. Whilst the introduction of EMHMTS was primarily aimed at increasing income earning options, it also contributed in a number of ways towards building SESR against climate change effects. Bridging social capital once again played an important role by providing technical know-how regarding the production and marketing of mangoes. One particularly important piece of knowledge that was acquired by peri-urban dwellers in the PK-SES is how to graft traditional mango species with the newer EMHMTS. Inherent within the knowledge transfer component of bridging social capital, this also has sustainability implications, as the acquired knowledge (technical know-how) can potentially have long-lasting benefits for the recipient SES—as was indeed the case in the PK-SES, several years after the program had been introduced. Like the bee keeping initiative, the EMHMTS strategy was also claimed to have contributed to reducing dependence on traditional provisioning ecosystem services in the PK-SES, thereby contributing to increased carbon storage and higher levels of carbon sequestration. At the same time, bridging social capital also played a role in promoting cultural ecosystem services (CES). Again, this was realized though synergies, integrating potential resources from within the PK-SES and external resources in the form of bridging social capital. This involved a number of the actors mentioned in Table 2: TFS and KDC on the side of the government and Afri Roots, Jane Goodall, Fly Company, PHCTE, KCTE, Safari 56, and MHCTE on the side of NGOs/private companies. In this realm of CES, the role of bridging social capital was manifested in several ways, first and foremost through the formation of some of the entities established for spearheading CES towards building SESR. In interview, an official of KDC shared the following:

The PK-SES has enormous CES potential for boosting the economy of peri-urban dwellers. This would in turn help to protect and safeguard the forest reserves thereby building resilience against climate change effects. Yet, in the past, there has been [very little] substantial effort deployed in tapping this opportunity for fruitful community and environmental impact. However, currently the KDC are giving particular attention to this area. In so doing, we have promoted and facilitated the formation of local cultural tourism based enterprises. These include: PHCTE, KCTE and the MHCTE. Whilst these local based cultural tourism enterprises are still in their infancy, they show signs of positive futures towards building a climate friendly local economy.

Another example of this role for bridging social capital is provided by Afri Roots. Whilst the PK-SES has numerous cultural tourism sites with potential for boosting climate friendly livelihoods among the peri-urban dwellers, these are largely underdeveloped (Mngumi 2019). It could be argued that the PK-SES had a resource deficit when it came to activating its huge, untapped cultural tourism potential, including a lack of technical know-how. However, through the bridging social capital provided by Afri Roots, infrastructure is now being improved with the aim of tapping the area’s cultural tourism potential. An Afri Roots representative stated in an interview that, despite having high cultural tourism potential, the PK-SES lacked the physical infrastructures that would encourage tourists to visit. Afri Roots had therefore supported the process of developing walkways to the tourist sites in the PK-SES, and had played a vital role in advertising the area’s cultural tourist attractions. Tourist numbers at the cultural tourism sites in the PK-SES have begun to climb. In addition, Afri Roots devised a 20 km weekend cycling tour from Dar es Salaam city to the cultural attraction sites of the PK-SES. This has further opened up the cultural tourism potential to local and international tourists. These interventions by a bridging social capital player (in this case Afri Roots) have also created climate friendly employment opportunities for peri-urban dwellers, thereby contributing to building SESR against climate change effects. Moreover, other interventions by Afri Roots in the PK-SES include promoting tourism related activities such as hiking, bird watching, mountain biking, and antiquities, as well as promoting Swahili culture and Swahili foods. Other bridging social capital players engaged in promoting cultural tourism include TFS, KDC, Safari 56, PHCTE, MHCTE and KCTE.

Another role played by bridging social capital in building resilience at the PK-SES was through promoting participatory forest management (PFM). This has been effected through integrated efforts between a number of social capital players, including WCST, TFS, KDC, TFCG, UMITASU, MJUMITA and Green Voices. There have been several initiatives geared at PFM: interventions in this area include promoting friendly relations between the communities in the forest reserve neighborhoods; encouraging climate friendly livelihood activities in the forest reserve and its neighborhoods, such as collecting forest products; and allowing community members to practice their traditional rituals and taboos within the forest reserves. PFM is argued to inculcate a spirit of environmental stewardship which in turn creates a sense of ownership and belonging and eventually reduces the level of forest encroachment, thereby boosting the forest’s carbon sequestration function. These claims are echoed by (Agrawal and Angelsen 2009). PFM, like other bridging social capital strategies mentioned, has contributed to building SESR against climate change effects though opening up climate friendly livelihood options.

However, in the course of building resilience to climate change effects at the PK-SES, one limitation facing the bridging social capital players (i.e. actors external to the PK-SES) was very clear. This is the problem of limited government support. The bridging social capital players have contributed substantially to the building of SESR in tandem with local players and resources. However, to make most effective use of this synergy with local communities in building SESR against climate change effects, the unstinting support of the government is of paramount importance. In this regard, the local government authority (KDC) and the central government agency responsible for safeguarding ecosystem services (TFS), had variously contributed towards building SESR as argued beforehand. However, during key informant interviews it was reported that government support is not as anticipated. The unqualified support from the government players was repeatedly emphasized during key informant interviews, as in the example below:

There is a tendency of ill-support from the government related players namely TFS and KDC. Despite we private players being willing to support and promote tourism industry in these peri-urban forest reserves, we have been receiving inadequate support from the government counterparts which is discouraging. There are a number of initiatives *(for instance creating walk ways along the forest reserves)* which we have volunteered to support the industry but surprisingly the government machinery instead of backing up our efforts turns out to be the stumbling block.

When a government representative responded to an inquiry regarding the inadequate support to bridging social capital players, a number of setbacks were mentioned. These included lack of manpower, unclear power relations, and budgetary limitations. Although not the central focus of this article, worries about sustainability also arose during key informant interviews. For example, several initiatives had been established aimed at building SESR for climate change effects as part of the Reducing Emissions from Deforestation and Degradation (REDD++) pilot project,^3^ but some of these are no longer functioning. It was noted that WCST, in particular, had made a substantial contribution in promoting and facilitating initiatives aimed at building resilience to climate change effects during the REDD++ project but at the end of the project, there was no effective sustainability plan. TFS was supposed to take over some of the initiatives started by WCST but to date it has not been able to fill that gap appropriately.

## Conclusions

The theoretical and empirical analysis presented in this paper on the potential contribution of social capital towards building SESR against climate change effects in peri-urban areas leads to two main conclusions. The first conclusion concerns the importance of social capital in building SESR in peri-urban areas of the developing world. The analysis has shown that both bonding and bridging social capital are essential ingredients in building peri-urban resilience. This argument aligns with current thinking that sees resilience primarily as a social phenomenon. Indeed, the social nature notio of resilience is even more relevant in urban and peri-urban areas in SSA, where engineering approaches has been largely dominant. This leads to the second conclusion from this research: that social capital needs to be clearly conceptualized and then factored into climate change resilience building pathways in peri-urban areas in SSA. This would in turn contribute to building resilient cities in the region, as peri-urban spaces constitute an integral yet largely neglected part of cities in SSA.

## Appendix

**Ardhi University**

**Institute of Human Settlements Studies**

**Socio-capital Survey on the PK-SES towards Building Resilience to Climate Change Effects**

**Part I: Basic demographic information**

- 1_Date of interview —/—/201—
- 2_Subward name——————
- 3_Ward name———————–
- 4_District name___ a) Kisarawe b) Ilala
- 5_Age of respondent/Household head___
- 6_Phone number of respondent________________
- 7_Residence status______ a) Native b) Migrant from Dar es Salaam c) Rural/Upcountry migrant
- 8_Sex of respondent___ a) Male b) Female
- 9_Education status____ a) Primary or less b) Secondary c) High school d) Certificate and above

**Part II: Social contact and people skills**

- 1_Among all the people you know, with how many do you interact well? ___ a) All b) Most c) Some d) Few e) None
- 2_How often do you have to deal with people in your work? ___ a) Always need to deal with people b) Most often need to deal with people c) Sometimes need to deal with people d) Rarely need to deal with people e) No need to deal with people
- 3_ Among all people who live in your community and the neighborhood, how many of them can support each other and get along with each other well?__ a) All b) Most c) Some d) Few e) None
- 4_Among all the governmental, political, economic, economic, social, cultural, recreational groups and organizations, how many can collaborate with each other? __ a) All b) Most c) Some d) Few e) None
- 5_How do you rate the frequency of doing the following activities?

**Table:**

Categories	Likert scale options				
	Every day	Often	Sometimes	Rarely	Never
Chatting with others	5	4	3	2	1
Gift giving	5	4	3	2	1
Working together	5	4	3	2	1
Playing together	5	4	3	2	1
Visiting each other	5	4	3	2	1
Communicating through telephone or internet	5	4	3	2	1
Offering assistance to others	5	4	3	2	1
Participating in parties and gatherings	5	4	3	2	1

**Part II: Social network (Bonding social capital)**

**Question One (Cap 1):** How do you rate the number of people in each of the following six categories

**Table:**

Categories	Likert scale options				
	A lot	More than average	Average	Less than Average	A few
Your family members	5	4	3	2	1
Your relatives	5	4	3	2	1
People in your neighborhood	5	4	3	2	1
Your friends	5	4	3	2	1
Your coworkers/fellows	5	4	3	2	1
Your country fellows/old classmates	5	4	3	2	1

Whereby A lot = > 10, More than average = (9-10), Average = (6-8), Less than average = (3-5), A few = (1-2).

**Question Two (Cap 2):** With how many people in each of the following categories do you keep a routine contact?

**Table:**

Categories	Likert scale options				
	All	Most	Some	Few	None
Your family members	5	4	3	2	1
Your relatives	5	4	3	2	1
People in your neighborhood	5	4	3	2	1
Your friends	5	4	3	2	1
Your coworkers/fellows	5	4	3	2	1
Your country fellows/old classmates	5	4	3	2	1

**Question Three (Cap 3):** Among the people in each of the following six categories, how many can you trust?

**Table:**

Categories	Likert scale options				
	All	Most	Some	Few	None
Your family members	5	4	3	2	1
Your relatives	5	4	3	2	1
People in your neighborhood	5	4	3	2	1
Your friends	5	4	3	2	1
Your coworkers/fellows	5	4	3	2	1
Your country fellows/old classmates	5	4	3	2	1

**Question Four (Cap 4):** Among people in each of the following six categories, how many will definitely help you upon your request?

**Table:**

Categories	Likert scale options				
	All	Most	Some	Few	None
Your family members	5	4	3	2	1
Your relatives	5	4	3	2	1
People in your neighborhood	5	4	3	2	1
Your friends	5	4	3	2	1
Your coworkers/fellows	5	4	3	2	1
Your country fellows/old classmates	5	4	3	2	1

**Question Five (Cap 5):** When people in all the six categories are considered, how many possess the following assets/resources?

**Table:**

Categories	Likert scale options				
	All	Most	Some	Few	None
Certain political power	5	4	3	2	1
Wealth or owners of an enterprise or a company	5	4	3	2	1
Broad connections with others	5	4	3	2	1
High reputation/influential	5	4	3	2	1
With high school or more education	5	4	3	2	1
With a professional job	5	4	3	2	1

**Part III: BRIDGING AND LINKING ISSUES**

**Question Six (Cap 6):** How do you rate the number of the following two types of groups/organizations in your community?

**Table:**

Categories	Likert scale options				
	A lot	More than average	Average	Less than average	A few
Governmental, political, economic and social groups/organizations (political parties, women’s groups, village communities, trade union, cooperate associations, volunteer groups, etc.)	5	4	3	2	1
Cultural, recreational and leisure groups/organizations (religious, country fellows, alumni, sport, music, dances, crafts, games, etc.)	5	4	3	2	1

**Question Seven (Cap 7):** Do you participate in activities for how many of each of these two types of groups and organizations?

**Table:**

Categories	Likert scale options				
	All	Most	Some	A few	None
Governmental, political, economic and social groups/organizations *(political parties, women’s groups, village communities, trade union, cooperate associations, volunteer groups,* etc.)	5	4	3	2	1
Cultural, recreational and leisure groups/organizations (*religious, country fellows, alumni, sport, music, dances, crafts, games,* etc*.)*	5	4	3	2	1

**Question Eight (Cap 8):** Among each of the two types of groups and organizations, how many represent your rights and interests?

**Table:**

Categories	Likert scale options				
	All	Most	Some	A few	None
Governmental, political, economic and social groups/organizations *(political parties, women’s groups, village communities, trade union, cooperate associations, volunteer groups,* etc.)	5	4	3	2	1
Cultural, recreational and leisure groups/organizations (*religious, country fellows, alumni, sport, music, dances, crafts, games,* etc*.)*	5	4	3	2	1

**Question Nine (Cap 9):** Among each of the two types of groups and organizations, how many will help you upon your request?

**Table:**

Categories	Likert scale options				
	All	Most	Some	A few	None
Governmental, political, economic and social groups/organizations *(political parties, women’s groups, village communities, trade union, cooperate associations, volunteer groups,* etc.)	5	4	3	2	1
Cultural, recreational and leisure groups/organizations (*religious, country fellows,alumni, sport, music, dances, crafts, games,* etc*.)*	5	4	3	2	1

**Question Ten (Cap 10):** When all groups and organizations in the two categories are considered, how many possess the following assets/resources?

**Table:**

Categories	Likert scale options				
	All	Most	Some	A few	None
Significant power for decision making	5	4	3	2	1
Solid financial basis	5	4	3	2	1
Broad connections with others	5	4	3	2	1
Broad social connections	5	4	3	2	1
Great social influence	5	4	3	2	1

**Question Eleven:** How would you describe the capacity of the Agent in team building in each of the specific leadership areas below?

**Table:**

Categories	Likert scale options				
	Very strong	Strong	Moderate	Little	None
Agent’s leadership ability of working together in building resilience to climate change effects	5	4	3	2	1
Agent’s ability to engage and include core actors in building resilience to climate change effects	5	4	3	2	1
Agent’s ability to share decision making and reach consensus with other actors	5	4	3	2	1
Agent’s ability to innovate, lead, inspire and keep resilience building actors unified (including grassroots)	5	4	3	2	1
Agent’s ability to develop working relationship with non-traditional allies	5	4	3	2	1
Agent’s ability to gain visibility and credibility with grassroots	5	4	3	2	1
Agent’s ability to gain visibility and credibility with higher level policy organs	5	4	3	2	1

## Footnotes

1 This refers to 10 items of the standard example questionnaire by Chen et al. The 10 items/Cap (identified as Cap 1–Cap 10) each comprise 42 sub-items, as presented in “Appendix”.

2 George and Mallery (2003) provide the following rule of thumb for Cronbach’s alpha scores: “≥ .9—Excellent, ≥ .8—Good, ≥ .7—Acceptable, ≥ .6—Questionable, ≥ .5—Poor, and ≤ .5 – Unacceptable.

3 PK-SES was the site of one of the REDD++ pilot projects in Tanzania from 2010 to 2015. Some SESR interventions were instituted by REDD++ projects.
